# Supplementary material for: Comparative Analysis of Re-Annotated Genes Provides Insight into Evolutionary Divergence and Expressions of Aquaporin Family in Pepper
Source: Plants (Basel). 2021 May 21;10(6):1039. doi: 10.3390/plants10061039 (PMC8224332; doi:10.3390/plants10061039)
Supplement: Supplementary file 1 [file plants-10-01039-s001.zip › 20210513_rev2_Plants_AQP_supplementary_figures_FINAL.pdf]

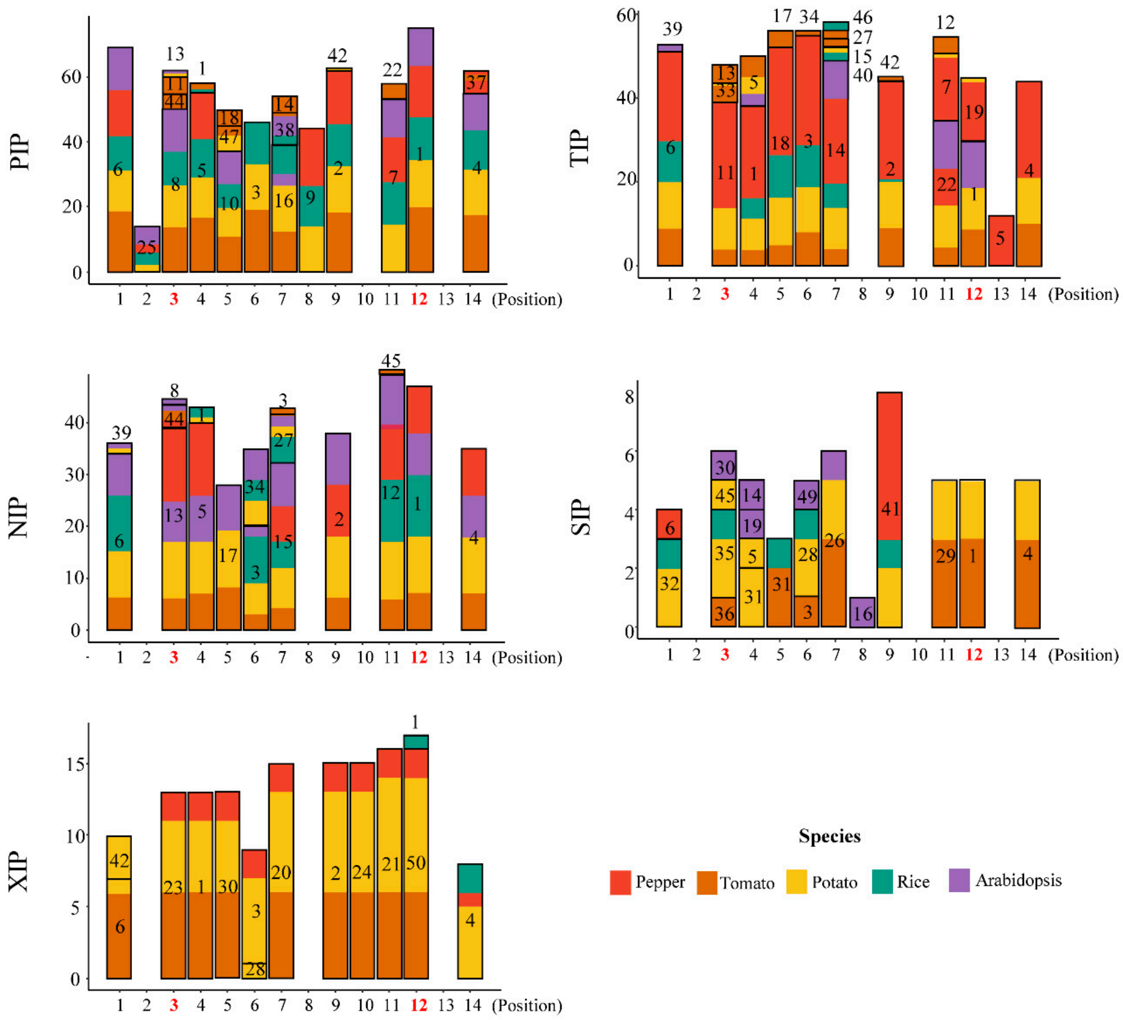

Supplementary Figure 1. Motif structures for aquaporin (AQP) domains of each subfamily in five plants species. Motif structures for aquaporin (AQP) domains of each subfamily five plant species. The AQP domains consist of a total of 14 positions, and positions highlighted in red represent the location of four residues of ar/R selectivity filter. The *y* -axes indicate the number of genes containing each motif. The motif numbers written in bars indicate motif sequences described in Table S3. Numbers of motifs in AQP domains of five plant species are shown as a bar graph. PIP, plasma membrane intrinsic protein subfamily; TIP, tonoplast intrinsic protein subfamily; NIP, NOD26-like intrinsic protein subfamily; SIP, small basic intrinsic protein subfamily; XIP, uncategorized X intrinsic protein subfamily.

A)

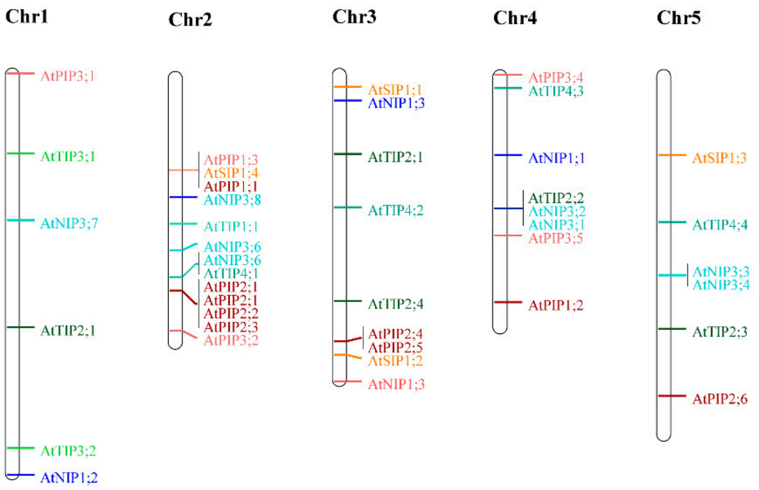
Chr1
Chr2
Chr3
Chr4
Chr5
AtPIP3;1
AtTIP3;1
AtNIP3;7
AtTIP2;1
AtTIP3;2
AtNIP1;2
AtPIP1;3
AtSIP1;4
AtPIP1;1
AtNIP3;8
AtTIP1;1
AtNIP3;6
AtNIP3;6
AtTIP4;1
AtPIP2;1
AtPIP2;1
AtPIP2;2
AtPIP2;3
AtPIP3;2
AtSIP1;1
AtNIP1;3
AtTIP2;1
AtTIP4;2
AtTIP2;4
AtPIP2;4
AtPIP2;5
AtSIP1;2
AtNIP1;3
AtPIP3;4
AtTIP4;3
AtNIP1;1
AtTIP2;2
AtNIP3;2
AtNIP3;1
AtPIP3;5
AtPIP1;2
AtSIP1;3
AtTIP4;4
AtNIP3;3
AtNIP3;4
AtTIP2;3
AtPIP2;6

B)

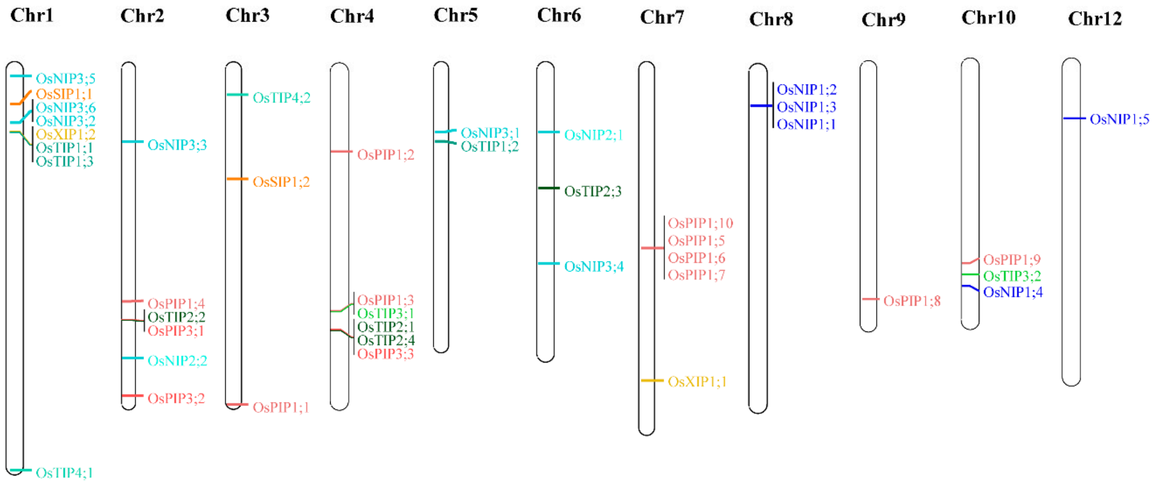
Chr1
Chr2
Chr3
Chr4
Chr5
Chr6
Chr7
Chr8
Chr9
Chr10
Chr12
OsNIP3;5
OsSIP1;1
OsNIP3;6
OsNIP3;2
OsXIP1;2
OsTIP1;1
OsTIP1;3
OsTIP4;1
OsNIP3;3
OsPIP1;4
OsTIP2;2
OsPIP3;1
OsNIP2;2
OsPIP3;2
OsTIP4;2
OsSIP1;2
OsPIP1;1
OsPIP1;2
OsPIP1;3
OsTIP3;1
OsTIP2;1
OsTIP2;4
OsPIP3;3
OsNIP3;1
OsTIP1;2
OsNIP2;1
OsTIP2;3
OsNIP3;4
OsPIP1;10
OsPIP1;5
OsPIP1;6
OsPIP1;7
OsXIP1;1
OsNIP1;2
OsNIP1;3
OsNIP1;1
OsPIP1;8
OsPIP1;9
OsTIP3;2
OsNIP1;4
OsNIP1;5

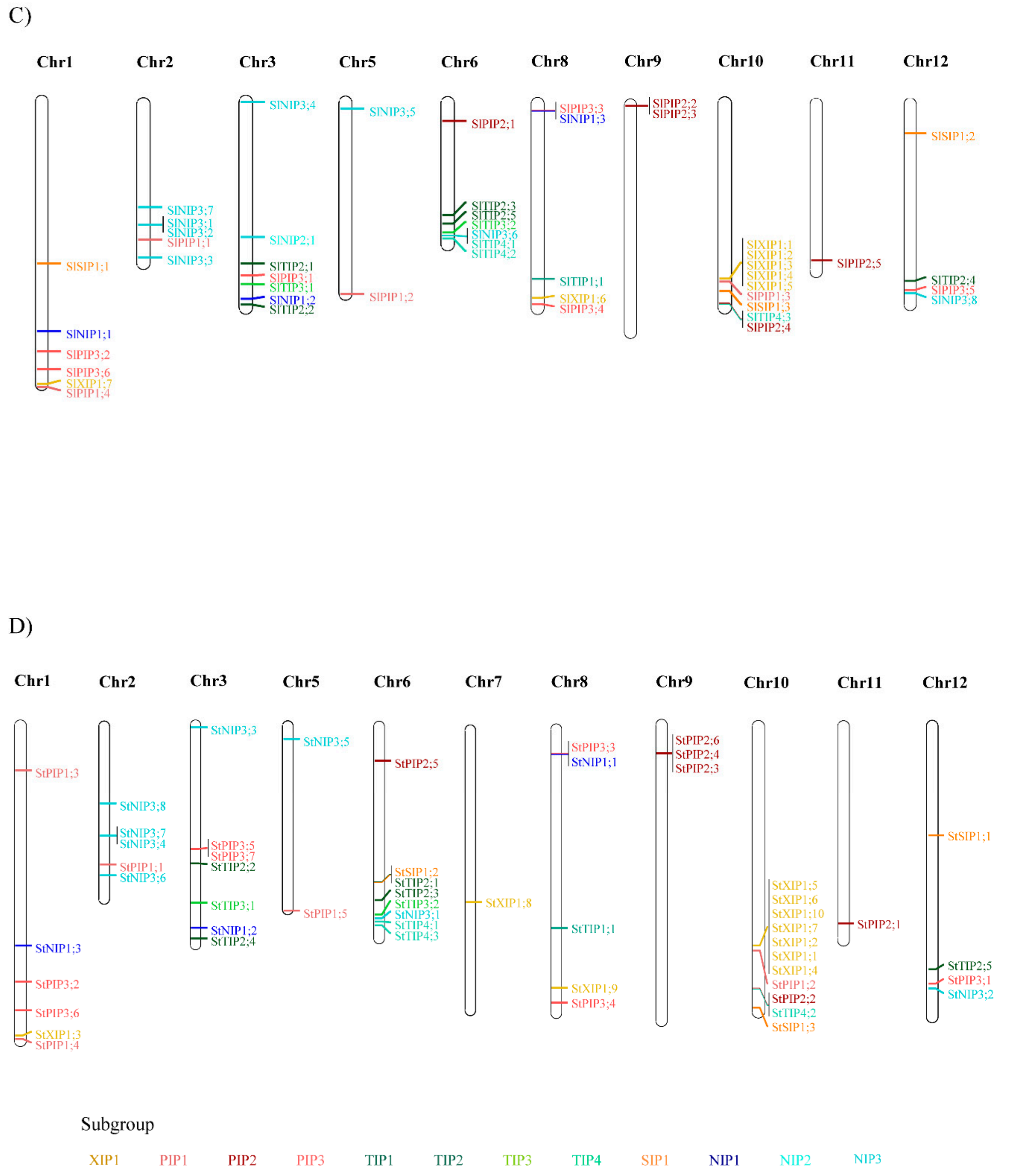

Supplementary Figure 2. Location of AQP genes on chromosomes for four plant species. AQP genes are shown for (A) *Arabidopsis,* (B) rice, (C) tomato, and (D) potato genomes. Colored gene names indicate the different subgroups. The number of AQP genes anchored to the chromosome was 41 Arabidopsis, 41 rice, 48 tomato, 56 potato and 64 pepper, respectively.

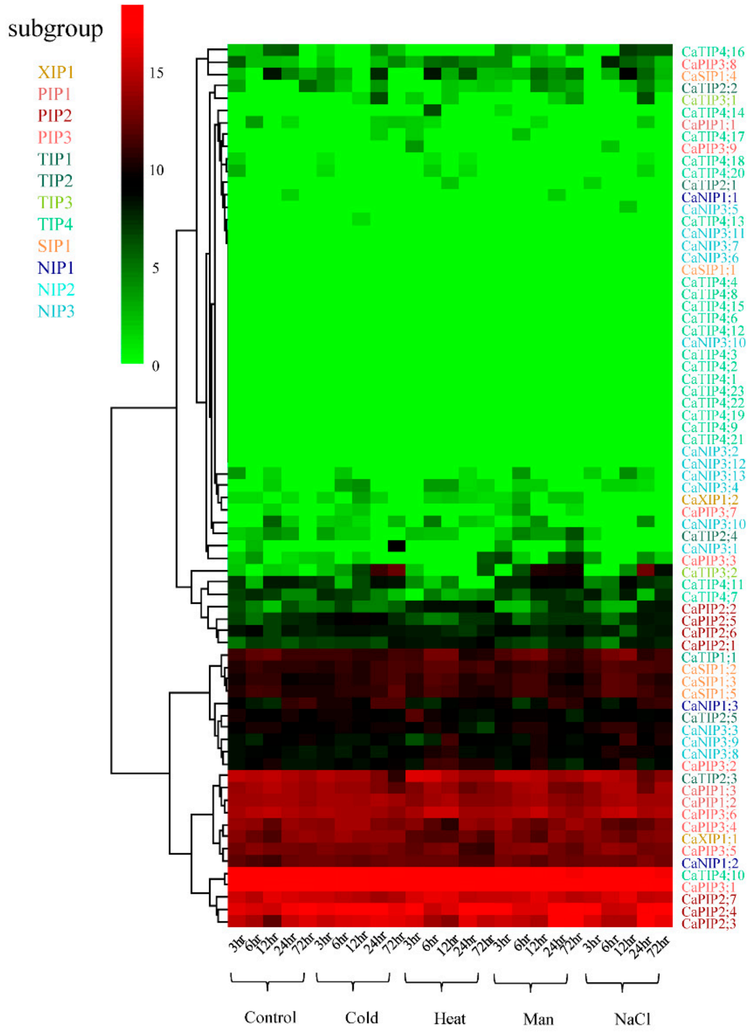

Supplementary Figure 3. Expression profiling of pepper AQP genes using RNA-Seq data under various abiotic stresses. Normalized FPKM values of pepper AQP genes are represented as a heatmap under different abiotic conditions. Colored scale shows a range of expression levels from 0 (green) to 20 (red). The color of each gene on the right represents the subgroups.
